# Poly[[bis­(μ-3-amino-5-carb­oxy­benzoato-κ^2^ *N*:*O* ^1^)diaqua­zinc] dihydrate]

**DOI:** 10.1107/S1600536812031789

**Published:** 2012-07-18

**Authors:** Kou-Lin Zhang, Ting-Ting Qiu, Seik Weng Ng

**Affiliations:** a College of Chemistry and Chemical Engineering, Yangzhou University, Yangzhou 225002, People’s Republic of China; b Department of Chemistry, University of Malaya, 50603 Kuala Lumpur, Malaysia; c Chemistry Department, King Abdulaziz University, PO Box 80203 Jeddah, Saudi Arabia

## Abstract

The Zn^II^ atom in the title polymeric compound, {[Zn(C_8_H_6_NO_4_)_2_(H_2_O)_2_]·2H_2_O}_*n*_, lies on a center of inversion and is coordinated by two amine N atoms and two carboxyl­ate O atoms from two 3-amino-5-carb­oxy­benzoate anions along with two water mol­ecules in a distorted octa­hedral geometry. The bridging nature of the anion generates a layer motif parallel to (100). Hydrogen bonds of the N—H⋯O and O—H⋯O types exist in the structure. One H atom of the coordinated water mol­ecule and one H atom of the solvent water mol­ecule are each disordered over two positions in a 1:1 ratio.

## Related literature

For *catena*-poly[(5-amino­isophthalato)aqua­zinc], see: Wu *et al.* (2002 ▶).
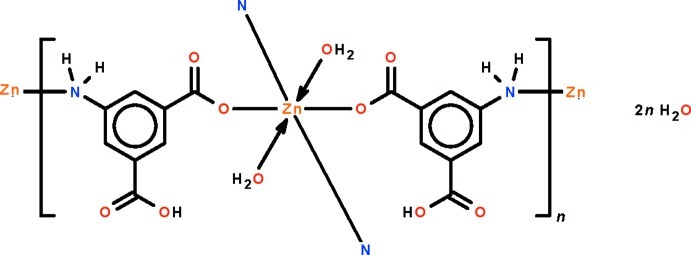


## Experimental

### 

#### Crystal data

[Zn(C_8_H_6_NO_4_)_2_(H_2_O)_2_]·2H_2_O
*M* *_r_* = 497.71
Monoclinic, 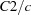

*a* = 14.2209 (10) Å
*b* = 11.2252 (8) Å
*c* = 12.7139 (9) Å
β = 113.286 (1)°
*V* = 1864.2 (2) Å^3^
*Z* = 4
Mo *K*α radiation
μ = 1.39 mm^−1^
*T* = 293 K
0.30 × 0.26 × 0.18 mm

#### Data collection

Bruker SMART APEX diffractometer
Absorption correction: multi-scan (*SADABS*; Sheldrick, 1996 ▶) *T*_min_ = 0.680, *T*_max_ = 0.788
8005 measured reflections
2130 independent reflections
1843 reflections with *I* > 2σ(*I*)
*R*_int_ = 0.018

#### Refinement

*R*[*F* ^2^ > 2σ(*F* ^2^)] = 0.034
*wR*(*F* ^2^) = 0.098
*S* = 1.07
2130 reflections
144 parameters
H-atom parameters constrained
Δρ_max_ = 0.37 e Å^−3^
Δρ_min_ = −0.37 e Å^−3^

### 

Data collection: *APEX2* (Bruker, 2005 ▶); cell refinement: *SAINT* (Bruker, 2005 ▶); data reduction: *SAINT*; program(s) used to solve structure: *SHELXS97* (Sheldrick, 2008 ▶); program(s) used to refine structure: *SHELXL97* (Sheldrick, 2008 ▶); molecular graphics: *OLEX* (Dolomanov *et al.*, 2003 ▶) and *X-SEED* (Barbour, 2001 ▶); software used to prepare material for publication: *publCIF* (Westrip, 2010 ▶).

## Supplementary Material

Crystal structure: contains datablock(s) global, I. DOI: 10.1107/S1600536812031789/xu5588sup1.cif

Structure factors: contains datablock(s) I. DOI: 10.1107/S1600536812031789/xu5588Isup2.hkl

Additional supplementary materials:  crystallographic information; 3D view; checkCIF report

## Figures and Tables

**Table 1:** Hydrogen-bond geometry (Å, °)

*D*—H⋯*A*	*D*—H	H⋯*A*	*D*⋯*A*	*D*—H⋯*A*
O3—H1⋯O2*W* ^i^	0.84	1.84	2.675 (3)	172
O1*W*—H2⋯O2^ii^	0.84	1.97	2.695 (3)	143
O2*W*—H4⋯O2	0.84	1.79	2.619 (3)	170
O2*W*—H5⋯O2*W* ^iii^	0.84	2.03	2.869 (5)	171
O2*W*—H5′⋯O1^iv^	0.84	2.31	3.106 (3)	158
O2*W*—H5′⋯O1*W* ^iv^	0.84	2.35	2.910 (3)	125
N1—H6⋯O4^v^	0.88	2.14	3.013 (3)	172
N1—H7⋯O4^vi^	0.88	2.21	3.059 (2)	161

Symmetry codes: (i) 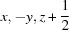; (ii) 
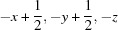
; (iii) 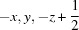; (iv) 
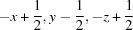
; (v) 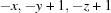; (vi) 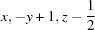.

**Fig. 1.**
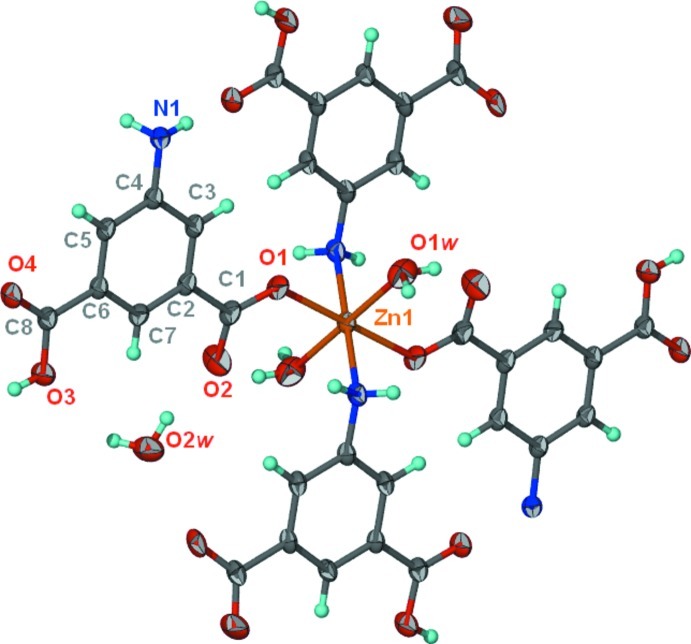
Thermal ellipsoid plot (Barbour, 2001) of a portion of polymeric Zn(H2O)2(C8H6NO4)2.2H2O at the 50% probability level; hydrogen atoms are drawn as spheres of arbitrary radius. The disorder in the hydrogen atoms of the coordinated and lattice water molecules is not shown.

**Fig. 2.**
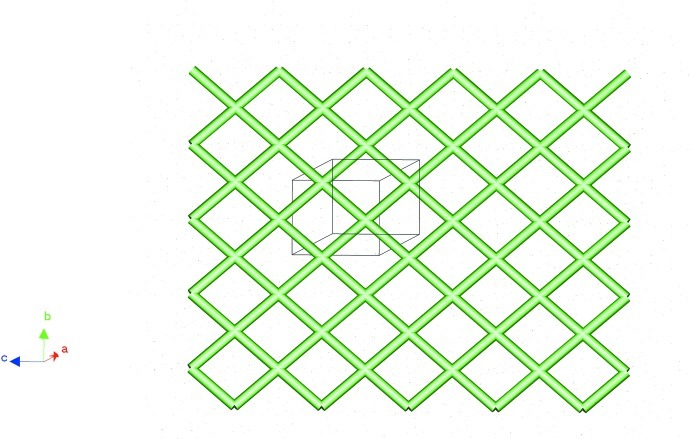
Layer motif represented as an OLEX-type (Dolomanov et al., 2003) of network.

## supplementary crystallographic information

### Comment

5-Aminoisophthalic acid furnishes a zinc derivative in which the ligand is dibasic; the dianion functions in a u3-bridging mode in monoaqua compound, which adopts a chain structure (Wu *et al.*, 2002). The title compound (Scheme I) was the unexpected when nicotinamide was added to the reaction.

The Zn^II^ atom in of polymeric [Zn(H_2_O)_2_(C_8_H_6_NO_4_)2^.^2H_2_O]*_n_* lies in a octahedron that is composed of two amino N atoms and two carboxyl O atoms (from two 5-aminoisophthalate anions) along with two water molecules (Fig. 1). The bridging nature of the anion generates a layer motif parallel to [1 0 0] (Fig. 2). Hydrogen bonds of the N–H···O and O–H···O type exist within the layer (Table 1).

### Experimental

5-Aminoisophthalic acid (0.030 g, 0.165 mmol) and sodium hydroxide (0.013 g, 0.330 mmol) were dissolved in a water/methanol (1:1 *v*/*v*; 10 ml) mixture. Zinc acetate hexahydrate (0.049 g, 0.165 mmol) dissolved in a water/methanol (1:1 *v*/*v*; 3 ml) mixture and nicotinamide (0.050 g, 0.33 mmol) dissolved in water (3 ml) were added. The solution was filtered. Light brown crystals were isolated after several days.

### Refinement

Carbon-bound H-atoms were placed in calculated positions (C—H 0.93 Å) and were included in the refinement in the riding model approximation, with *U*(H) set to 1.2*U*(*C*). The amino, water and acid H atoms were similarly positioned [N–H 0.88, O–H 0.84 Å; *U*(H) 1.2–1.5*U*(*N*,*O*)].

One H-atom of the coordinated water molecule and one H-atom of the lattice water molecule are each disordered over two positions in a 1:1 ratio. In the disorder model, the unprimed H-atoms are not within 2 Å of the primed ones.

### Crystal data

**Table:**

[Zn(C_8_H_6_NO_4_)_2_(H_2_O)_2_]·2H_2_O	*F*(000) = 1024
*M**_r_* = 497.71	*D*_x_ = 1.773 Mg m^−^^3^
Monoclinic, *C*2/*c*	Mo *K*α radiation, λ = 0.71073 Å
Hall symbol: -C 2yc	Cell parameters from 4815 reflections
*a* = 14.2209 (10) Å	θ = 2.4–28.2°
*b* = 11.2252 (8) Å	µ = 1.39 mm^−^^1^
*c* = 12.7139 (9) Å	*T* = 293 K
β = 113.286 (1)°	Prism, light brown
*V* = 1864.2 (2) Å^3^	0.30 × 0.26 × 0.18 mm
*Z* = 4	

### Data collection

**Table:**

Bruker SMART APEX diffractometer	2130 independent reflections
Radiation source: fine-focus sealed tube	1843 reflections with *I* > 2σ(*I*)
Graphite monochromator	*R*_int_ = 0.018
ω scans	θ_max_ = 27.5°, θ_min_ = 2.4°
Absorption correction: multi-scan (*SADABS*; Sheldrick, 1996)	*h* = −18→15
*T*_min_ = 0.680, *T*_max_ = 0.788	*k* = −14→14
8005 measured reflections	*l* = −16→16

### Refinement

**Table:**

Refinement on *F*^2^	Primary atom site location: structure-invariant direct methods
Least-squares matrix: full	Secondary atom site location: difference Fourier map
*R*[*F*^2^ > 2σ(*F*^2^)] = 0.034	Hydrogen site location: inferred from neighbouring sites
*wR*(*F*^2^) = 0.098	H-atom parameters constrained
*S* = 1.07	*w* = 1/[σ^2^(*F*_o_^2^) + (0.0506*P*)^2^ + 3.2779*P*] where *P* = (*F*_o_^2^ + 2*F*_c_^2^)/3
2130 reflections	(Δ/σ)_max_ = 0.001
144 parameters	Δρ_max_ = 0.37 e Å^−^^3^
0 restraints	Δρ_min_ = −0.37 e Å^−^^3^

### Fractional atomic coordinates and isotropic or equivalent isotropic displacement parameters (Å^2^)

**Table:**

	*x*	*y*	*z*	*U*_iso_*/*U*_eq_	Occ. (<1)
Zn1	0.2500	0.2500	0.0000	0.02950 (14)	
O1	0.20751 (15)	0.32463 (16)	0.12770 (14)	0.0412 (4)	
O2	0.1529 (2)	0.1571 (2)	0.1754 (2)	0.0655 (7)	
O3	0.09628 (17)	0.14472 (16)	0.53610 (16)	0.0436 (4)	
H1	0.0885	0.1154	0.5929	0.065*	
O4	0.07472 (15)	0.31377 (16)	0.61500 (15)	0.0408 (4)	
O1W	0.35790 (16)	0.38916 (19)	0.03707 (18)	0.0531 (5)	
H2	0.3743	0.3994	−0.0189	0.080*	
H3	0.4102	0.3714	0.0953	0.080*	0.50
H3'	0.3322	0.4522	0.0496	0.080*	0.50
O2W	0.0898 (2)	−0.04635 (18)	0.22475 (18)	0.0594 (6)	
H4	0.1052	0.0229	0.2118	0.089*	
H5	0.0334	−0.0439	0.2320	0.089*	0.50
H5'	0.1397	−0.0755	0.2797	0.089*	0.50
N1	0.12864 (15)	0.64309 (17)	0.36983 (16)	0.0312 (4)	
H6	0.0705	0.6633	0.3742	0.037*	
H7	0.1250	0.6675	0.3025	0.037*	
C1	0.1718 (2)	0.2655 (2)	0.1878 (2)	0.0349 (5)	
C2	0.15207 (17)	0.3304 (2)	0.28176 (18)	0.0291 (4)	
C3	0.15228 (17)	0.4538 (2)	0.28397 (18)	0.0294 (5)	
H3A	0.1667	0.4963	0.2293	0.035*	
C4	0.13087 (16)	0.5152 (2)	0.36787 (17)	0.0274 (4)	
C5	0.11166 (17)	0.4506 (2)	0.45058 (18)	0.0295 (5)	
H5A	0.0982	0.4904	0.5073	0.035*	
C6	0.11247 (17)	0.3261 (2)	0.44894 (17)	0.0277 (4)	
C7	0.13167 (19)	0.2662 (2)	0.3638 (2)	0.0302 (5)	
H7A	0.1309	0.1834	0.3618	0.036*	
C8	0.09264 (19)	0.2626 (2)	0.54063 (19)	0.0309 (5)	

### Atomic displacement parameters (Å^2^)

**Table:**

	*U*^11^	*U*^22^	*U*^33^	*U*^12^	*U*^13^	*U*^23^
Zn1	0.0412 (2)	0.0297 (2)	0.0248 (2)	−0.00028 (15)	0.02079 (16)	−0.00161 (13)
O1	0.0631 (12)	0.0436 (10)	0.0312 (9)	0.0127 (9)	0.0339 (8)	0.0038 (7)
O2	0.121 (2)	0.0466 (12)	0.0583 (13)	−0.0156 (13)	0.0664 (14)	−0.0190 (10)
O3	0.0725 (13)	0.0354 (9)	0.0375 (9)	0.0045 (9)	0.0374 (9)	0.0025 (7)
O4	0.0639 (12)	0.0404 (10)	0.0321 (9)	−0.0022 (9)	0.0340 (8)	−0.0024 (7)
O1W	0.0501 (11)	0.0531 (12)	0.0538 (12)	−0.0046 (9)	0.0181 (9)	−0.0207 (10)
O2W	0.1023 (18)	0.0426 (11)	0.0469 (11)	0.0111 (11)	0.0441 (12)	0.0074 (9)
N1	0.0419 (11)	0.0334 (10)	0.0242 (9)	0.0046 (8)	0.0194 (8)	0.0030 (7)
C1	0.0436 (13)	0.0414 (14)	0.0258 (11)	0.0043 (10)	0.0203 (10)	−0.0048 (9)
C2	0.0323 (11)	0.0388 (12)	0.0199 (9)	0.0037 (9)	0.0142 (8)	−0.0027 (8)
C3	0.0324 (11)	0.0394 (12)	0.0203 (9)	0.0035 (9)	0.0148 (8)	0.0011 (8)
C4	0.0290 (10)	0.0343 (11)	0.0208 (9)	0.0016 (9)	0.0118 (8)	−0.0011 (8)
C5	0.0340 (11)	0.0367 (12)	0.0227 (10)	0.0037 (9)	0.0165 (9)	−0.0024 (8)
C6	0.0295 (11)	0.0366 (11)	0.0200 (9)	0.0015 (9)	0.0129 (8)	0.0000 (8)
C7	0.0376 (12)	0.0323 (11)	0.0256 (10)	0.0033 (9)	0.0178 (9)	−0.0022 (8)
C8	0.0356 (11)	0.0380 (12)	0.0233 (10)	0.0028 (9)	0.0160 (9)	0.0013 (8)

### Geometric parameters (Å, º)

**Table:**

Zn1—O1W	2.108 (2)	O2W—H5'	0.8407
Zn1—O1W^i^	2.108 (2)	N1—C4	1.436 (3)
Zn1—O1	2.1163 (16)	N1—Zn1^iv^	2.214 (2)
Zn1—O1^i^	2.1163 (16)	N1—H6	0.8800
Zn1—N1^ii^	2.214 (2)	N1—H7	0.8800
Zn1—N1^iii^	2.214 (2)	C1—C2	1.517 (3)
O1—C1	1.261 (3)	C2—C7	1.390 (3)
O2—C1	1.243 (3)	C2—C3	1.386 (3)
O3—C8	1.326 (3)	C3—C4	1.401 (3)
O3—H1	0.8400	C3—H3A	0.9300
O4—C8	1.217 (3)	C4—C5	1.390 (3)
O1W—H2	0.8400	C5—C6	1.398 (3)
O1W—H3	0.8400	C5—H5A	0.9300
O1W—H3'	0.8400	C6—C7	1.390 (3)
O2W—H4	0.8408	C6—C8	1.486 (3)
O2W—H5	0.8425	C7—H7A	0.9300
			
O1W—Zn1—O1W^i^	180.00 (9)	Zn1^iv^—N1—H6	106.7
O1W—Zn1—O1	86.47 (8)	C4—N1—H7	106.7
O1W^i^—Zn1—O1	93.53 (8)	Zn1^iv^—N1—H7	106.7
O1W—Zn1—O1^i^	93.53 (8)	H6—N1—H7	106.6
O1W^i^—Zn1—O1^i^	86.47 (8)	O2—C1—O1	123.9 (2)
O1—Zn1—O1^i^	180.00 (8)	O2—C1—C2	118.2 (2)
O1W—Zn1—N1^ii^	92.80 (8)	O1—C1—C2	117.9 (2)
O1W^i^—Zn1—N1^ii^	87.20 (8)	C7—C2—C3	120.2 (2)
O1—Zn1—N1^ii^	89.10 (7)	C7—C2—C1	120.1 (2)
O1^i^—Zn1—N1^ii^	90.90 (7)	C3—C2—C1	119.8 (2)
O1W—Zn1—N1^iii^	87.20 (8)	C2—C3—C4	120.5 (2)
O1W^i^—Zn1—N1^iii^	92.80 (8)	C2—C3—H3A	119.7
O1—Zn1—N1^iii^	90.90 (7)	C4—C3—H3A	119.7
O1^i^—Zn1—N1^iii^	89.10 (7)	C5—C4—C3	119.1 (2)
N1^ii^—Zn1—N1^iii^	180.00 (18)	C5—C4—N1	119.72 (19)
C1—O1—Zn1	124.19 (16)	C3—C4—N1	121.18 (19)
C8—O3—H1	109.5	C4—C5—C6	120.31 (19)
Zn1—O1W—H2	109.5	C4—C5—H5A	119.8
Zn1—O1W—H3	109.5	C6—C5—H5A	119.8
H2—O1W—H3	109.5	C7—C6—C5	120.1 (2)
Zn1—O1W—H3'	109.5	C7—C6—C8	122.4 (2)
H2—O1W—H3'	109.5	C5—C6—C8	117.58 (19)
H3—O1W—H3'	109.5	C2—C7—C6	119.8 (2)
H4—O2W—H5	108.9	C2—C7—H7A	120.1
H4—O2W—H5'	108.8	C6—C7—H7A	120.1
H5—O2W—H5'	116.7	O4—C8—O3	121.9 (2)
C4—N1—Zn1^iv^	122.60 (14)	O4—C8—C6	123.1 (2)
C4—N1—H6	106.7	O3—C8—C6	114.96 (19)
			
O1W—Zn1—O1—C1	−150.9 (2)	Zn1^iv^—N1—C4—C5	72.4 (2)
O1W^i^—Zn1—O1—C1	29.1 (2)	Zn1^iv^—N1—C4—C3	−107.5 (2)
N1^ii^—Zn1—O1—C1	116.2 (2)	C3—C4—C5—C6	−0.8 (3)
N1^iii^—Zn1—O1—C1	−63.8 (2)	N1—C4—C5—C6	179.3 (2)
Zn1—O1—C1—O2	−3.4 (4)	C4—C5—C6—C7	−0.6 (3)
Zn1—O1—C1—C2	175.68 (15)	C4—C5—C6—C8	179.3 (2)
O2—C1—C2—C7	11.5 (4)	C3—C2—C7—C6	−0.7 (3)
O1—C1—C2—C7	−167.7 (2)	C1—C2—C7—C6	−179.2 (2)
O2—C1—C2—C3	−167.1 (3)	C5—C6—C7—C2	1.3 (3)
O1—C1—C2—C3	13.8 (3)	C8—C6—C7—C2	−178.5 (2)
C7—C2—C3—C4	−0.7 (3)	C7—C6—C8—O4	−179.2 (2)
C1—C2—C3—C4	177.9 (2)	C5—C6—C8—O4	1.0 (3)
C2—C3—C4—C5	1.4 (3)	C7—C6—C8—O3	1.0 (3)
C2—C3—C4—N1	−178.7 (2)	C5—C6—C8—O3	−178.8 (2)

Symmetry codes: (i) −*x*+1/2, −*y*+1/2, −*z*; (ii) *x*, −*y*+1, *z*−1/2; (iii) −*x*+1/2, *y*−1/2, −*z*+1/2; (iv) −*x*+1/2, *y*+1/2, −*z*+1/2.

### Hydrogen-bond geometry (Å, º)

**Table:**

*D*—H···*A*	*D*—H	H···*A*	*D*···*A*	*D*—H···*A*
O3—H1···O2*W*^v^	0.84	1.84	2.675 (3)	172
O1*W*—H2···O2^i^	0.84	1.97	2.695 (3)	143
O2*W*—H4···O2	0.84	1.79	2.619 (3)	170
O2*W*—H5···O2*W*^vi^	0.84	2.03	2.869 (5)	171
O2*W*—H5′···O1^iii^	0.84	2.31	3.106 (3)	158
O2*W*—H5′···O1*W*^iii^	0.84	2.35	2.910 (3)	125
N1—H6···O4^vii^	0.88	2.14	3.013 (3)	172
N1—H7···O4^ii^	0.88	2.21	3.059 (2)	161

Symmetry codes: (i) −*x*+1/2, −*y*+1/2, −*z*; (ii) *x*, −*y*+1, *z*−1/2; (iii) −*x*+1/2, *y*−1/2, −*z*+1/2; (v) *x*, −*y*, *z*+1/2; (vi) −*x*, *y*, −*z*+1/2; (vii) −*x*, −*y*+1, −*z*+1.

## References

[bb1] Barbour, L. J. (2001). *J. Supramol. Chem.* **1**, 189–191.

[bb2] Bruker (2005). *APEX2* and *SAINT* Bruker AXS Inc., Madison, Wisconsin, USA.

[bb3] Dolomanov, O. V., Blake, A. J., Champness, N. R. & Schröder, M. (2003). *J. Appl. Cryst.* **36**, 1283–1284.

[bb4] Sheldrick, G. M. (1996). *SADABS* University of Göttingen, Germany.

[bb5] Sheldrick, G. M. (2008). *Acta Cryst.* A**64**, 112–122. 10.1107/S0108767307043930 18156677

[bb6] Westrip, S. P. (2010). *J. Appl. Cryst.* **43**, 920–925.

[bb7] Wu, C.-D., Lu, C.-Z., Yang, W.-B., Zhuang, H.-H. & Huang, J.-S. (2002). *Inorg. Chem.* **41**, 3302–3307. 10.1021/ic011182g 12055009

